# Rapid Intravenous Glyceryl Trinitrate in Ischemic Damage (RIGID) After Stroke: Rationale, Design and Protocol for a Prospective Randomized Controlled Trial

**DOI:** 10.3389/fneur.2021.693330

**Published:** 2021-08-04

**Authors:** Lipeng Cai, Gary Rajah, Honglian Duan, Jie Gao, Zhe Cheng, Ruiqiang Xin, Shangqian Jiang, Peter Palmer, Xiaokun Geng, Yuchuan Ding

**Affiliations:** ^1^Department of Neurology, Beijing Luhe Hospital, Capital Medical University, Beijing, China; ^2^Department of Neurosurgery, Munson Medical Center, Traverse City, MI, United States; ^3^Department of Medical Imaging, Luhe Hospital, Capital Medical University, Beijing, China; ^4^Department of China-America Institute of Neuroscience, Beijing Luhe Hospital, Capital Medical University, Beijing, China; ^5^Department of Neurology, Northeast Ohio Medical University, Rootstown, OH, United States; ^6^Department of Neurosurgery, Wayne State University School of Medicine, Detroit, MI, United States

**Keywords:** acute ischemic stroke, neuroprotection, nitric oxide, glyceryl trinitrate (GTN), intravenous thrombolysis

## Abstract

**Background:** Despite intravenous thrombolysis and endovascular therapy for acute ischemic stroke (AIS), many survivors still have varying degrees of disability. Glyceryl trinitrate (GTN), a nitric oxide (NO) donor, has been previously reported to induce neuroprotection after AIS. The use of GTN to reduce brain damage after stroke remains yet to be elucidated. This study was designed to explore the safety, feasibility, and preliminary efficacy of intravenous administration of GTN after AIS.

**Methods:** A prospective randomized controlled trial is proposed with AIS patients. Participants will be randomly allocated to GTN group and control group with a 1:1 ratio (*n* = 40). Both groups will be treated with standard therapies according to the current stroke guidelines. Participants allocated to the GTN group will receive intravenous administration of GTN (5 mg GTN in 50 ml saline at a rate of 0.4 mg/h that is continued for 12.5 h/day for 2 days) within 24 h of symptom onset. Participants allocated to the control group will receive intravenous administration at equal capacity of 0.9% normal saline (NS) (total 50 ml/day at 4 ml/h that is continued for 12.5 h/day for 2 days). The primary outcome is safety [systolic blood pressure (SBP) <110 mmHg, headache], while the secondary outcomes include changes in functional outcome and infarction volume.

**Discussion:** Rapid Intravenous Glyceryl Trinitrate in Ischemic Damage (RIGID) is a prospective randomized controlled trial that aims to ascertain the safety, feasibility, and preliminary efficacy of intravenous GTN as a neuroprotection strategy after AIS. These results will provide parameters for future studies as well as provide insights into treatment effects. Any possible neuroprotective qualities of GTN in AIS will also be elucidated.

**Trial Registration:**www.chictr.org.cn, identifier: ChiCTR2100046271.

## Introduction

Due to its high rate of mortality and morbidity, ischemic stroke is a devastating public health concern that also results in high socioeconomic burden (1–4). The effective treatments for acute ischemic stroke (AIS) are intravenous thrombolysis and mechanical thrombectomy. Due to the narrow time window for intravenous alteplase [tissue plasminogen activator (tPA)] of 4.5 h, the majority of patients are not eligible for this treatment. In addition, two-thirds of stroke patients still suffer from varying degrees of disability (5, 6) even after intravenous thrombolysis. Endovascular therapy (within 6 h of stroke and up to 24 h) has shown great benefit in improving functional outcomes in AIS patients with large vessel occluded (LVO) in the anterior circulation. However, only 46% of the patients achieve functional independence at 90 days with a 15.3% mortality rate (7). In the EXTEND trial (8), patients benefited from tPA between 4.5 and 9.0 h after the onset of stroke. This study suggested that the “tissue window” has advantages over the traditional “time window” in screening patients. Thus, exploring fast and effective neuroprotection strategies to save ischemic penumbra and to lengthen the “tissue window” may be the key in the treatment of AIS.

Ischemic stroke-induced brain damage results from the interaction of complex pathophysiological processes such as excitotoxicity, oxidative stress, inflammation, and apoptosis (9). NO has a central role in hypoxic signaling, and its physiologic and therapeutic levels exert potent cytoprotection after ischemia and reperfusion in various tissues including the brain (10). NO derived from endothelial nitric oxide synthase (eNOS) plays a critical role in the regulation of cerebral microvascular tone, the protection of the blood–brain barrier, the reduction of oxidative stress, and the alleviation of procoagulant stimulation (11–13). Various animal studies have reliably demonstrated a loss of cytoprotection when subjects were treated with NO scavengers or when NOS was inhibited or knocked out (14–16). NO donors are a heterogeneous group of drugs whose common feature is the ability to release NO or an NO-related species *in vitro* or *in vivo* independently of endogenous sources (17). NO donors have been implicated in improving cancer therapy, hypertension, and peripheral artery disease (17). Several preclinical studies suggest that NO donors could safely reduce infarct size, increase cerebral blood flow, and improve functional outcome in AIS in both transient and permanent stroke models (18). The neuroprotective effect of NO donors has been previously demonstrated to work at many different levels by several mechanisms including that of altering the cellular oxidative status, inhibiting monocyte activity, and diminishing primary hemostasis (19).

GTN, a Food and Drug Administration-approved vasodilator, is an example of a drug that functions as a NO donor. Transdermal GTN had been found to lower blood pressure (BP), have no deleterious effects on platelet function, and exert no changes in the middle cerebral artery blood flow velocity or regional cerebral blood flow in AIS patients (20–23). A recent larger sample size randomized controlled trial (RCT) to determine the efficacy of transdermal GTN for the management of high BP in AIS (ENOS) also revealed the potential to reduce BP without finding a functional improvement following AIS if administered within 48 h (24). However, this study indicated that administration of GTN within 6 h improved functional outcomes. The RIGHT-2 trial (25) focused on the safety and efficacy of transdermal GTN given within 4 h of onset of AIS assessed in the prehospital environment in the UK. This trial did not show that prehospital treatment with transdermal GTN improved functional outcomes in patients with presumed stroke. Importantly, the neuroprotective effect of NO donors after ischemia–reperfusion injury (IRI) has yet to be reported consistently with many factors contributing to this including dose, location, source, and environment (26). Further protocols have been proposed to evaluate the use of transdermal GTN in acute stroke treatment (26). In light of these parameters and owing to the short half-life of GTN, GTN administration as a patch on the arm or chest may not reach an effective concentration in the cerebrovascular system. As such, a continuous intravenous administration of GTN may be a rapid and effective way to maximize any benefit of this drug (26). Furthermore, 24-h continuous GTN administration can cause tolerance resulting in subtherapeutic levels. This knowledge hints that a better outcome may be possible if an “intermittent” therapy is used (27–29). At present, no study has been reported on the safety and efficacy of intravenous GTN as an adjuvant neuroprotective strategy for AIS. We have consequently designed this single-center, prospective RCT to evaluate the safety, feasibility, and preliminary efficacy of intravenous administration of GTN after AIS.

## Methods

### Study Design

This study is a phase 1, single-center, prospective RCT. Participants will be patients with AIS within 24 h onset. Patients meeting the inclusion criteria but not the exclusion criteria will be randomly allocated to the GTN group or the control group. Both groups will be treated with the standard management according to the guidelines (30). GTN will be administered by continuous intravenous pump (5 mg GTN in 50 ml saline with a speed of GTN 0.4 mg/h continued for 12.5 h/day for 2 days) within 24 h of symptom onset in the GTN group. The control group will receive intravenous administration of equal capacity of 0.9% normal saline (NS) (total 50 ml/day at 4 ml/h continued for 12.5 h/day for 2 days). The clinical characteristics, medical history (diabetes, hypertension, hyperlipidemia, coronary heart disease, stroke, atrial fibrillation), smoking history, alcohol drinking history, and National Institutes of Health Stroke Scale (NIHSS) score at admission will be collected. Magnetic resonance imaging (MRI) will be performed at baseline and on day 7 ± 1. NIHSS and modified Rankin Scale (mRS) will be assessed at baseline and on days 1, 7, 14, 30, and 90.

All participants or proxies will be informed of potential risks and possible benefits and consent to this study. This consent will be provided to a legal representative if the patients do not have the capacity to consent. This study was approved by the ethics committee of Luhe Hospital, Capital Medical University, Beijing, China, and has been registered at www.chictr.org.cn with ChiCTR2100046271. An independent physician will monitor the health and safety of the participants.

### Patient Population: Inclusion and Exclusion Criteria

**Figure 1:**
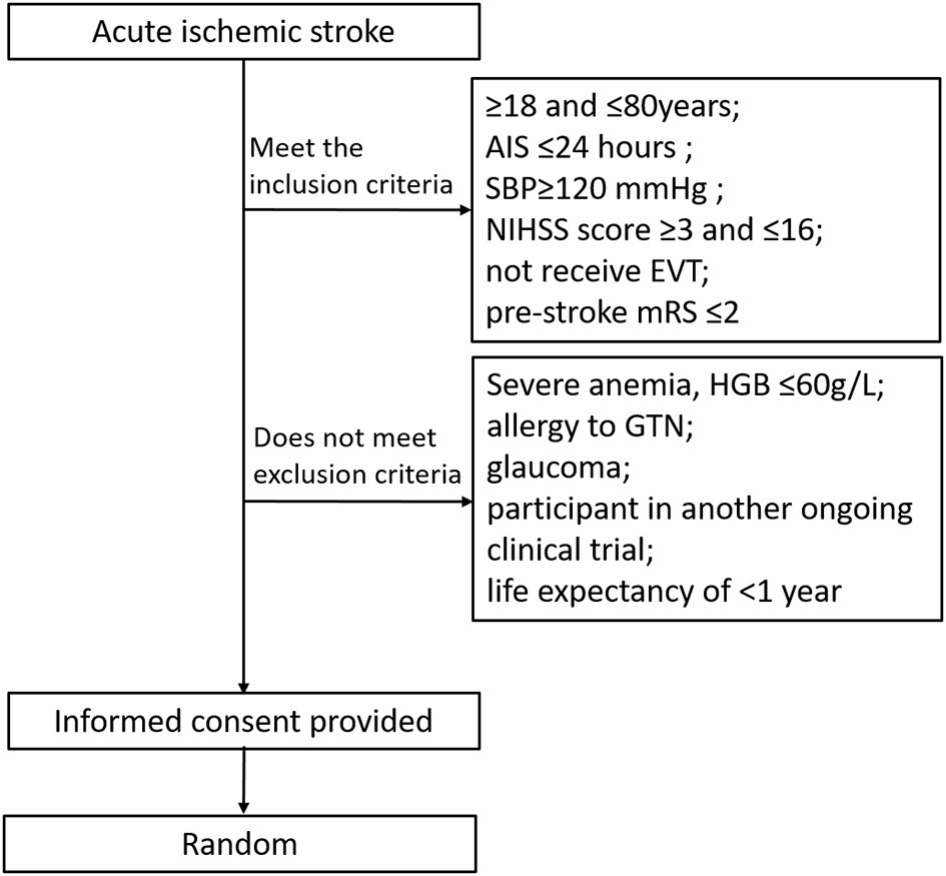
Trial randomization flowchart. AIS, acute ischemic stroke; NIHSS, national institutes of health stroke scale; SBP, systolic blood pressure; mRS, modified rankin scale; GTN, glycerly trinitrate; HGB, hemoglobin.

Participants will be recruited from the stroke center [the Stroke Intervention & Translational Center (SITC)] in Beijing Luhe Hospital (Figure 1). The inclusion criteria are (1) ≥18 and ≤ 80 years old, (2) clinical diagnosis of AIS, (3) systolic blood pressure (SBP) ≥120 mmHg, (4) NIHSS score ≥3 and ≤ 16, (5) patients with time from onset to treatment ≤ 24 h who did not receive endovascular treatment (EVT), (6) prestroke mRS ≤ 2, and (7) informed consent provided by participant or legally authorized representative.

Exclusion criteria are as follows: (1) severe anemia, hemoglobin (HGB) 60 g/L, (2) allergy to GTN, (3) glaucoma, (4) participant in another ongoing clinical trial, and (5) life expectancy of shorter than 1 year due to comorbidities.

### Randomization and Blindness

During the recruitment period, participants will be allocated 1:1 to two groups (*n* = 40) by computer-generated randomization procedures using opaque envelopes. A research assistant not involved in the study will prepare the envelopes before the study. After recording baseline measures, participants will be randomly allocated to either the intervention or the control group by the treating physicians, who will open the sealed opaque envelopes. In order to minimize selection bias, patients and assessors involved in the trial will be masked to the treatment allocation. All outcome measurements will be assessed by two observers who will be blinded to the treatment plan. Any disagreement will be resolved by reaching a consensus between the two. If no consensus can be reached, a third observer blinded to the treatment assignment and not involved in the clinical treatment plan will have the final decision. Finally, an independent investigator blinded to the treatment assignment will collect the data of outcomes and information of the group and analyze them.

### Interventions

Participants in both groups will be treated with the standard management according to the guidelines (30). In order to ensure the stability of administration speed and the stability of BP, patients allocated to the GTN group will undergo intravenous administration of GTN (5 mg GTN in 50 ml saline, with a speed of GTN 0.4 mg/h continued for 12.5 h/day, for 2 days) within 24 h of symptom onset. In the control group, 0.9% NS will be administered by continuous intravenous pumping with a speed of 4 ml/h continued for 12.5 h/day, for 2 days. Since no standard intravenous dose of GTN is available for AIS, the doses and administration of intravenous GTN are determined by following considerations. First, according to routine dosage of intravenous GTN, 0.3 mg/h (up to 20 mg/h) is applied. Second, because of the dose-dependent reductions in SBP (31) by GTN, we will use the similar low dose at 0.4 mg/h for 12.5 h/day to prevent excessive reduction of systemic BP for safety purposes. In addition, compared with intravenous administration, studies (20, 21, 25, 32) have shown that transdermal GTN patches (at a 0.4 mg/h rate for 12.5 h during a single application), in which there are about 75% of nitroglycerin systemically bioavailable after administration (33), reduce SBP by 5.8–13 mmHg after AIS that is within the range in the present study. Vital signs (i.e., BP, heart rate, body temperature, respiratory rate of the patients) and possible adverse drug reactions such as hypotension and headache, will be closely monitored during the whole treatment period. If the patients show a tendency to develop adverse reactions and complications, the trial shall be immediately stopped, and routine treatment shall be given.

### Outcomes

#### Primary Outcomes (Safety Assessment)

The primary safety outcome is SBP <110 mmHg. The primary outcome SBP <110 mmHg was defined as average SBP <110 mmHg within 24 h after GTN has been started. BP will be measured every 15 min between 0 and 2 h after GTN is started, every 30 min between 2 and 12 h, every 120 min between 12 and 48 h, and twice a day after 48 h. The target levels of BP poststroke remain unclear within the available literature (34–37). The current AIS guidelines differentiate BP targets based on a variety of factors including whether the patient receives alteplase, undergoes mechanical embolectomy, and/or experiences a hemorrhagic conversion. Prior studies (34–37) found a U-shaped relation of BP with functional outcome: both low BP and high BP were associated with poor outcome. There is no clearly defined cutoff for low BP in patients with AIS. Previously identified nadirs such as the tipping point in the U-shaped association between BP and outcome also vary between 120 and 180 mmHg (34, 36). Because of this variability, according to prior studies, we will use the lowest 10th percentile as a cutoff, low SBP, namely, SBP <110 mmHg.

The secondary safety outcomes are headache. Headaches related to GTN were defined as follows: GTN responders are those who develop a mild to moderate headache (headache scores 3–6) within 5–15 min with a short-lasting duration (maximum of 30 min) and spontaneously recover within 1 h after administration of GTN without the need for any rescue medication (38). In addition, severe headaches (scores 7–10) or the use of analgesia for GTN-caused headaches will be accounted for as secondary safety outcomes. If patients experience severe headaches, vital signs (i.e., BP, heart rate, body temperature, respiratory rate of the patients) will be closely monitored, and a CT scan will be obtained to analyze the cause of the headache.

#### Secondary Outcomes (Efficacy Assessment)

The primary efficacy outcome is the mRS at 90 days (mRS scores of 0–2 indicate functional independence). Secondary efficacy outcomes include the rate of the 0–2 mRS at 90 days, the incidence of death at 90 days, blood nitrate index detection at 1 day [cyclic guanosine monophosphate (cGMP, second messenger to NO), L-arginine (substrate for NO), and L-citrulline (co-product with NO)], infarct volume, as well as NIHSS scores at days 1, 7, 14, 30, and 90. The mRS and NIHSS scores will be obtained by a blinded personnel to the research. Face-to-face encounters in the consultation room will be used in the study to calculate mRS and NIHSS. Brain infarct volume will be determined by MRI diffusion-weighted imaging technique. The lesion profile plotted at each individual level by an image tool [region of interest (ROI)] on the workstation will be used to calculate the area. The levels will be multiplied by the thickness of each level and summed to calculate the infarct volume. The calculations will be performed by personnel blinded to clinical data and randomization at baseline and at day 7 ± 1.

Patients diagnosed as AIS without corresponding lesions on MRI are rare (<2%) in our stroke center. Patients who are misdiagnosed with stroke through negative MRI will be reported, and subgroup analysis will be performed.

### Estimation of Sample Size

There are no data available for reference because no completed clinical study of intravenous GTN in AIS patients currently exists. However, Hertzog (39) has suggested that 10–20 patients in each group are sufficient to assess the feasibility of a pilot study, while Dobkin (40) has shown that 15 patients in each group is usually enough to decide whether a larger multicenter trial should be conducted. In order to determine the sample size for each group, a power analysis was conducted based on the work of a prior study (20) that GTN can make a difference of 10 mmHg in SBP as compared to placebo groups. For the difference in BP at 10 mmHg, standard deviation at 10 mmHg, in order to have alpha exceed 95%, and beta = 0.8, a sample size of 16 patients per group has been calculated. As we expect to include around 20% for treatment dropouts and crossovers and for losses to follow-up, we will increase this sample size with 20% and aim to recruit 40 patients. The results of this study should be able to determine the initial safety and feasibility of intravenous infusion of GTN in AIS patients. The data will be used to estimate sample size and conduct a power calculation to plan a phase 2 trial for efficacy.

### Statistical Analyses

All analyses are based on the intention-to-treat (ITT) principle, including all randomly enrolled subjects. Compared to the control group, if the GTN group did not have an increase in the incidence of adverse reactions and there was no difference in 90-day prognosis between the two groups, we would move forward with a phase 2 trial.

Categorical variables including the proportion of good functional outcomes and the frequency of adverse events will be presented as counts and percentages. χ^2^ test, Fisher exact test, or continuity correction will be used where appropriate for comparison between the two groups. If the continuous variables including NIHSS score and infarct volume conform to the normal distribution, results shall be indicated by mean ± standard deviation and tested by *t*-test, and if they do not conform to the normal distribution, results shall be indicated by median and interquartile range and tested by Mann–Whitney *U*-test. p < 0.05 will be considered statistically significant. SPSS 22.0 software (IBM Inc., Armonk, NY) will be used for statistical analysis.

## Discussion

Over the past few decades, over 1,000 neuroprotective methods have been examined for adjunct neuroprotective administration in the setting of AIS (41). However, most neuroprotectants have only demonstrated benefit in animal stroke models without successful clinical transformation (42, 43). The cause of this failure was not immediately clear. One general conclusion would be that there is a low likelihood of success in targeting one pathway or one selective mechanism within a rather heterogeneous, nongenetically determined condition operative over many years (44). Ischemic stroke-induced brain injury results from the interaction of complex pathophysiological processes such as excitotoxicity, oxidative stress, inflammation, and apoptosis (9, 45–47). The post-ischemic cascade is a complex, multipathway, multifactorial process involving a variety of pathological mechanisms; therefore, multiple targets of neuroprotection drugs may be more effective.

NO is a ubiquitous molecule in the body, which plays a multitude of physiological actions such as a vasodilator, neurotransmitter, immunomodulator, and antagonist of platelets and leukocytes (48). In the brain, NO is mainly synthesized by various subtypes of NOSs: neuronal nitric oxide synthase (nNOS), eNOS, and inducible nitric oxide synthase (iNOS) (25). Previous studies demonstrate that organ injury can occur due to a reduction of NO, most commonly due to a reduction in eNOS activity during ischemia reperfusion injury (IRI) (49). In the setting of IRI, NO has been found to have various protective effects on inhibiting oxidative stress, leukocyte–endothelial adhesion, cytokine release, and apoptosis (50). NO can also reduce infarct size and inflammation after ischemic stroke and improve cerebral blood flow, cerebral metabolism, and nerve function in a preclinical study (51). A previous observational study found that patients with ischemic stroke had significantly lower plasma NO levels than matched normal volunteers, and the low NO levels were associated with more severe stroke and worse outcome assessed at discharge disposition (52). A meta-analysis showed a significant association between different eNOS gene polymorphisms and risk of ischemic stroke in the Asian population (52).

NO plays a pivotal role in preventing inflammation and attenuating oxidative stress after IRI, and, therefore, NO supplementation using NO donor drugs is a reasonable approach to minimizing the cerebral damage after ischemia. GTN is one of the most widely used exogenous NO donors in the clinic. Three studies (20–22) on transdermal GTN in acute stroke showed that GTN lowered peripheral and central BP, 24-h BP, pulse pressure, and augmentation index. However, RCTs ENOS (53) and RIGHT-2 (25) on transdermal GTN patch (5 mg) showed that there was no significant change in functional or secondary outcomes measured at day 90. In a prespecified subgroup analysis of participants within 6 h of stroke presentation in the ENOS trial, those who received GTN had a favorable improvement in functional outcomes, less death and disability, and improved cognition (25). Transdermal GTN is a simple method to the delivery of GTN; however, not all of the drug released from the patch reaches the systemic circulation (54). Compared with intravenous administration, about 75% of nitroglycerin is systemically bioavailable after patch administration (55). The reason for lost drug is related to retention at the application site, tissue binding, and breakdown (33). The development of an intravenous form of nitroglycerin has further enhanced the role of nitrates in the therapy of cardiovascular disorders. Intravenous form of nitroglycerin permits prompt initiation of therapy and rapid attainment of high systemic levels (56). Although there is concern that lowering BP may worsen outcomes in the context of carotid stenosis, an analysis of the ENOS trial demonstrated that transdermal GTN appeared safe in both ipsilateral and bilateral stenoses (25). Considering that both GTN and NO (57) have a very short half-life in the body, an intravenous form of nitroglycerin with rapid dose titration is both feasible and safe. This may be the best method in a clinical trial for stroke patients to receive targeted cerebral NO donors. Prior clinical trials on the continuous application of nitroglycerin patches showed that 24-h continued use of GTN results in developing tolerance in the majority of patients with stable angina and suggested that “intermittent” therapy may provide a more rational approach to therapy. With removal of the patch for 10–12 h in each 24-h period, this will provide a patch-free period, which may allow the reestablishment of sensitivity (27–29). The daily dose at 5 mg/day [0.4 mg/h (54)] was recommended in the two important RCTs ENOS and RIGHT-2 on transdermal GTN patch (25, 58). In order to rescue the ischemic penumbra that infarct completed within 48 h after stroke onset (59), GTN at a speed of GTN 0.4 mg/h for 12.5 h/day, which makes 5 mg/day in total, was used in the present study, for 2 days. We will determine whether intravenous GTN is safe and has the potential to improve functional outcomes in patients with AIS.

There are limitations to this study. First, this is a single-center, small-sample experiment, which may affect the generalizability of the interventions. Second, although the target dosage has been shown to be safe and reliable in other small-cohort experiments, the dose used in the present study may still need optimization. In addition, there is a concern that an intravenous administration of an equal dosage of GTN might have more interactions given better bioavailability.

Rapid Intravenous Glyceryl Trinitrate in Ischemic Damage (RIGID) is designed to identify the safety, feasibility, and possible efficacy of intravenous administration of GTN in AIS patients. The preliminary results will provide clues for the design of future clinical trials. Based on past basic research and previous clinical studies, we predict that intravenous administration of GTN is safe for patients with AIS. The current proposed study may suggest a neuroprotective role for GTN in AIS and, thus, warrants an RCT.

## Ethics Statement

The studies involving human participants were reviewed and approved by the Ethics Committee of Beijing Luhe Hospital, Capital Medical University, Beijing, China. The patients/participants provided their written informed consent to participate in this study.

## Author Contributions

YD and XG conceptualized the study and contributed to the study design and implementation. LC made substantial contributions to the design, implementation, and writing of the protocol. GR and PP contributed to the design of the trial from their area of expertise. HD, JG, ZC, RX, and SJ contributed to the implementation of specific procedures, conceptualized the study and contributed to the study design and implementation. All authors contributed to the article and approved the submitted version.

## Conflict of Interest

The authors declare that the research was conducted in the absence of any commercial or financial relationships that could be construed as a potential conflict of interest.

## Publisher's Note

All claims expressed in this article are solely those of the authors and do not necessarily represent those of their affiliated organizations, or those of the publisher, the editors and the reviewers. Any product that may be evaluated in this article, or claim that may be made by its manufacturer, is not guaranteed or endorsed by the publisher.
